# Assessment of the Annual Additional Effective Doses amongst Minamisoma Children during the Second Year after the Fukushima Daiichi Nuclear Power Plant Disaster

**DOI:** 10.1371/journal.pone.0129114

**Published:** 2015-06-08

**Authors:** Masaharu Tsubokura, Shigeaki Kato, Tomohiro Morita, Shuhei Nomura, Masahiro Kami, Kikugoro Sakaihara, Tatsuo Hanai, Tomoyoshi Oikawa, Yukio Kanazawa

**Affiliations:** 1 Division of Social Communication System for Advanced Clinical Research, Institute of Medical Science, University of Tokyo, Minato-ku, Tokyo, Japan; 2 Department of Radiation Protection, Minamisoma Municipal General Hospital, Minamisoma, Fukushima, Japan; 3 Department of Radiation Protection, Soma Central Hospital, Soma, Fukushima, Japan; 4 Department of Epidemiology and Biostatistics, School of Public Health, Imperial College London, Norfolk Place, London, United Kingdom; University of South Carolina, UNITED STATES

## Abstract

An assessment of the external and internal radiation exposure levels, which includes calculation of effective doses from chronic radiation exposure and assessment of long-term radiation-related health risks, has become mandatory for residents living near the nuclear power plant in Fukushima, Japan. Data for all primary and secondary children in Minamisoma who participated in both external and internal screening programs were employed to assess the annual additional effective dose acquired due to the Fukushima Daiichi nuclear power plant disaster. In total, 881 children took part in both internal and external radiation exposure screening programs between 1^st^ April 2012 to 31^st^ March 2013. The level of additional effective doses ranged from 0.025 to 3.49 mSv/year with the median of 0.70 mSv/year. While 99.7% of the children (n = 878) were not detected with internal contamination, 90.3% of the additional effective doses was the result of external radiation exposure. This finding is relatively consistent with the doses estimated by the United Nations Scientific Committee on the Effects of Atomic Radiation (UNSCEAR). The present study showed that the level of annual additional effective doses among children in Minamisoma has been low, even after the inter-individual differences were taken into account. The dose from internal radiation exposure was negligible presumably due to the success of contaminated food control.

## Introduction

Radiation exposure can result in potential long-term health risks, such as increased incidence of tumor, depending on effective doses.[1] It is known that health threats emerged in radiation-contaminated areas after nuclear accidents such as the Chernobyl disaster.[2] Cumulative radiation exposure has become a serious public concern in Fukushima, particularly for children as they have a higher chance of developing health issues in the future related to the nuclear power plant disaster.[3, 4] Chronic radiation exposure constitutes a substantial fraction of long-term cumulative radiation exposure among residents in radiation-contaminated areas due to prolonged radiation contamination in the surrounding environment, including soil and locally grown produce, as was the case after the Chernobyl disaster.[5, 6] Therefore, reducing doses from chronic radiation exposure is important for managing public health of people living in and near the affected areas.[7]

Doses from chronic radiation exposure can be classified into two types: external and internal radiation exposures. The level of external and internal radiation can be estimated using the data on soil and food contamination as demonstrated in publications by the World Health Organization (WHO)[8] and the United Nations Scientific Committee on the Effects of Atomic Radiation (UNSCEAR).[9] For example, the level of external and internal radiation exposure through inhalation can be estimated using the data on surface radioactivity density, and the level of internal radiation exposure through ingestion can be calculated using the data on food radionuclide concentrations.

However, methodologies for dose estimation have several uncertainties associated with incomplete knowledge and information.[10] Estimated doses from several authorities tend to be higher than the actual dose measurements due to conservative hypotheses.[8] In addition, such estimations calculate representative effective doses of an average resident without considering inter-individual differences in radiation exposure and their resulting doses.[11, 12] Previous studies reported that both external and internal doses varied substantially between individuals according to their lifestyles, even among residents living in the same radiation-contaminated area.[13] Therefore, an individual assessment of doses is indispensable for accurate evaluation of health risks for each resident.

**Fig 1:**
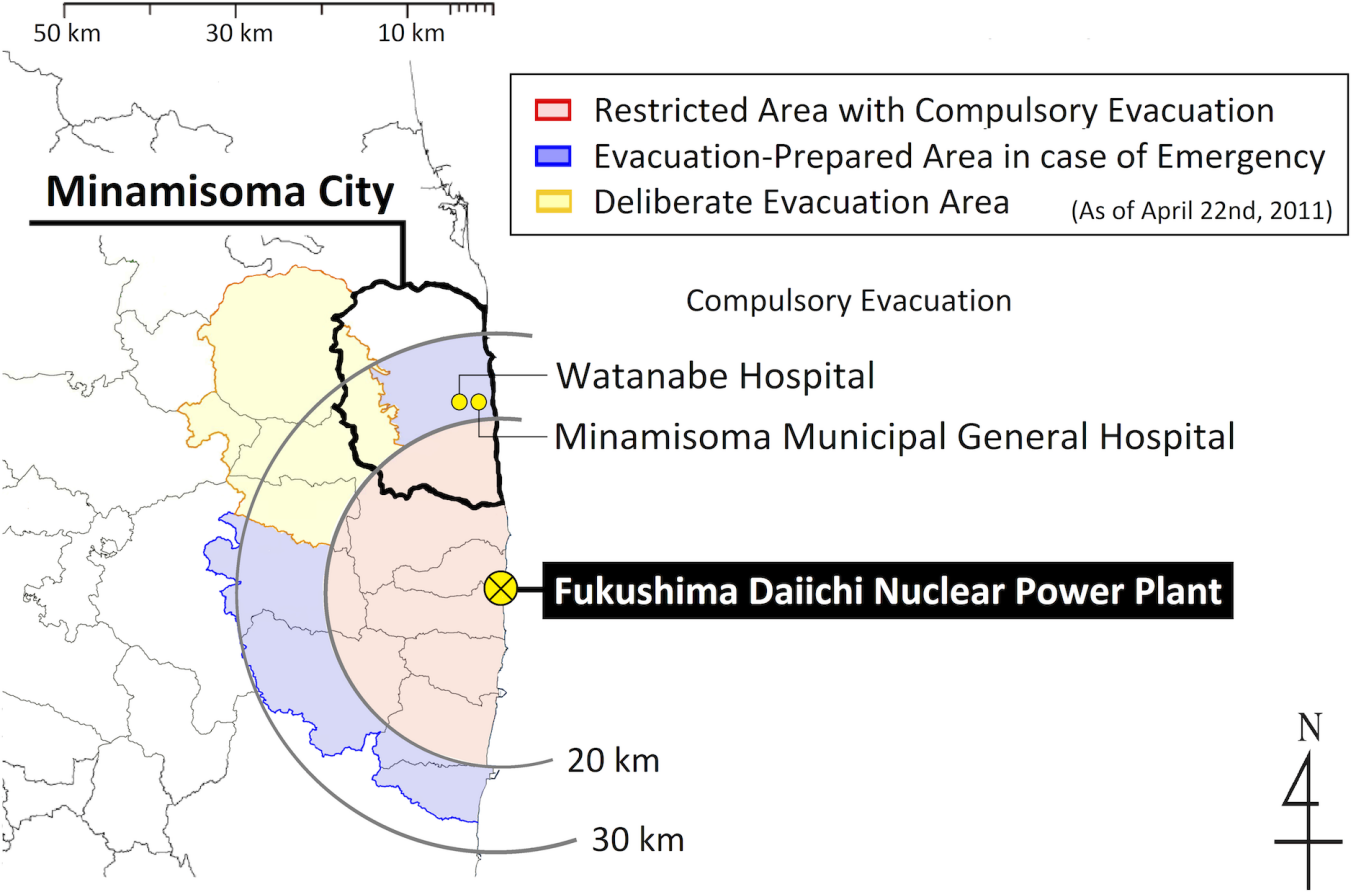
Location information of Minamisoma city. All WBC measurements were performed at Minamisoma Municipal General Hospital and Watanabe Hospital, both of which are located 23 km north of the Fukushima Daiichi nuclear power plant.

In response to the Fukushima Daiichi nuclear power plant disaster, which occurred on March 11^th^, 2011, several local municipalities in the Fukushima prefecture have independently performed an internal radiation exposure evaluation.[14, 15] Minamisoma, a highly contaminated coastal city located 14–38 km north of the power plant, has three designated evacuation zones which were declared by the Japanese government on April 22^nd^, 2011.[16] (Fig 1) In July 2011, the first local government-led screening program after the Fukushima Daiichi nuclear power plant disaster begun in Minamisoma as an internal radiation exposure screening program using a whole body counter (WBC).

Previous studies showed that chronic internal contamination was absent from all primary and secondary students in Minamisoma.[17] However, a little is known about the level of chronic external radiation exposure among this population. Since exposure pathways and countermeasures between external and internal radiation exposure are different, it is not possible to extrapolate internal to external effective doses from the data.

Balonov et al. reported that the proportions of doses from external and internal radiation exposure that account for the total effective dose after the Chernobyl disaster were approximately 60% and 40%, respectively.[18] However due to the limited data, it has not been possible to calculate the ratio of internal to external radiation exposure after the Fukushima Daiichi nuclear disaster. Thus, an evaluation of individual external radiation exposure has become mandatory for estimating the total chronic effective doses (i.e. the sum of doses from external and internal radiation exposure) which may cause long-term health risks.

In October 2011, Minamisoma launched a voluntary external radiation exposure screening program in collaboration with Chiyoda Technol Corp., Japan, a manufacturer of personalized radiation monitoring services and devices. Since then, repetitive measurements of external radiation exposure among children have been performed. In order to evaluate the levels of chronic radiation exposure among residents near the Fukushima Daiichi nuclear power plant and to clarify inter-individual differences of doses, we examined individual annual additional effective doses among children in Minamisoma, where periodic, consistent internal and external radiation monitoring has been performed.

## Materials and Methods

Data was collected on the primary and secondary students in Minamisoma who voluntary participated in both external and internal screening programs between 1^st^ April 2012 and 31^st^ March 2013. From the database, data on age, sex, and the results of external and internal screening programs during the study period were extracted. The annual additional effective doses due to external and internal radiation exposure were calculated respectively, and were combined. A distribution of the proportion of doses attributed to external exposure among the participants was visualized using histogram charts and a univariate kernel density estimation (a non-parametric way to estimate the probability density function of a random variable) based on a Gaussian kernel (i.e., the standard normal density function) with an optimal bandwidth following Silverman’s rule.

### Internal radiation exposure screening program

Internal radiation contamination levels among elementary and middle school students from 22 schools located within Minamisoma were assessed during the study period. The WBC measurements conducted between 1^st^ April 2012 and 31^st^ March 2013 at the Minamisoma Municipal General Hospital and the Watanabe Hospital were used. Main variables examined were age, sex, and the total body burden of radioactive Cesium (Cs: Cs-134 and Cs-137) as an indicator of the total level of internal radiation exposure.[19] To reduce the risk of bias of the results through contaminated clothing, all children were required to change into a gown before the WBC measurements.

### Test procedure

Two hospitals in the city retain a permanent WBC system. The WBC machine at the Minamisoma Municipal General Hospital is a stereoscopic machine with two 3 x 5 x 16 cubic inch NaI scintillation detectors (Fastscan Model 2251; Canberra, Inc., Meriden, CT, USA). A 30-cm stool was used for students shorter than 130 cm. The WBC machine in the Watanabe hospital is chair-shaped and has two 3 x 5 x 16 cubic inch NaI scintillation detectors (WBC-R43-22458; Hitachi Aloka Medical, Ltd., Mitaka, Tokyo, Japan). The detection limits of both machines following a two-minute scan are 220 Bq/body for Cs-134 and 250 Bq/body for Cs-137. A team at the National Institute of Radiological Sciences checked the WBCs using four sets of Bottle MAnnikin ABsorber (BOMAB) phantoms (Co-60, Cs-137, Ba-133 and water, manufactured by the Japan Radioisotope Association), and the overall efficiency of the detectors was accurate within 10%.

### Effective dose calculation for internal radiation exposure

Annual effective doses from the internal contamination of ingested Cs-134 and Cs-137 were evaluated on the basis of the effective dose coefficients derived from the International Commission on Radiological Protection, Publication 67.[20] An assumption was made that the amount of Cs activity detected at the WBC examinations was in an equilibrium state between consecutive ingestions and excretions for a year. The effective dose coefficients for Cs-134 and Cs-137 depend on the ages of the subjects.

### External radiation exposure screening program

This program is free of charge for infants, children and pregnant women who are registered in the Minamisoma family registry. Notification of the program was sent to schools, and the information was also disseminated using a city magazine distributed to each household.

The screening program has been conducted every three months. First, the city office sent a radiation dosemeter (Glass Badge: GD-450, Chiyoda Technol Corp.) to individuals or students’ guardians who gave a consent to participate in the program. Next, screenings were performed 3 times between 1^st^ April 2012 to 31^st^ March 2013 (1st screening: 1^st^ June until 31^st^ August 2012; 2^nd^ screening: 1^st^ September until 30^th^ November 2012; and 3^rd^ screening 1^st^ December 2012 until 28^th^ February 2013). During the screening, the participants were instructed to always carry a dosemeter, which measure their doses from external radiation exposure for three months. Then, the dosemeters were returned to the city office, and the Minamisoma Municipal General Hospital records the measured doses of external radiation exposure.

To calculate additional effective doses from external radiation exposure, the dose from natural sources including the universe (cosmic rays from the sun) and compounds within the earth need to be subtracted from the measured value. The values to be subtracted were set by Chiyoda Technol Corp. as 0.54 mSv/year. They were the average values of the 20 Glass Badges with a 35 days measurement time placed at Oarai, Ibaraki Prefecture, Japan, before the disaster. The range of measurement accuracy of the cumulative dose within 35 days was ± 4%.

The result of the Glass Badge measurement is expressed as a dose equivalent at a tissue depth of 1 cm (Hp 10). Hp (10) values obtained in the conditions of the affected areas in Fukushima Prefecture are known to be comparable with the effective dose of isotropic (ISO) or rotation (ROT) irradiation geometries.[21–23] Thus, we regarded the Glass Badge-measured dose to be equivalent to an effective dose in the present study.

### Effective dose calculation for external radiation exposure

To express annual additional effective doses from external radiation exposure, data from students who participated in the external screening program for three times between 1^st^ April 2012 and 31^st^ March 2013 was used (1st screening: 1^st^ June until 31^st^ August 2012; 2^nd^ screening: 1^st^ September until 30^th^ November 2012; and 3^rd^ screening 1^st^ December 2012 until 28^th^ February 2013). Data from the three study periods were added. To calculate the dose per day, the combined result was divided by the total measurement period. Annual additional effective doses from external radiation exposure were calculated by multiplying this by 365.

### Ethics

The Institutional Review Board of University of Tokyo approved the study (authorization number 25-40-1011). For the use of internal contamination data, written informed consent was obtained from participants, their guardians or caretakers on behalf of the children enrolled in this study. For the use of external exposure data, the ethics committee has agreed that a written consent is not required to be obtained from each participant.

## Results

In total of 987 students participated in both internal and external radiation exposure screening programs between 1^st^ April 2012 and 31^st^ March 2013. After excluding those who were living outside of Minamisoma at the time of the screenings (n = 106), 881 students were left for the analysis. This number comprised 30% of the children aged 6–15 years in Minamisoma as of 6^th^ April 2012. The median age of the participants was 10, and 456 of the participants (approximately 52%) were female. 840 out of 881 children have experienced evacuations and returned to Minamisoma during the early phase of the disaster.

### Annual Additional Effective Dose from internal radiation exposure

Three students were detected with an internal Cs contamination: the levels of Cs-134 and Cs-137 for each of the student were [245; 566], [364; 452], and [401; 615] Bq/body, respectively. These values correspond to doses of 0.040, 0.069, and 0.085 mSv/year, respectively. 878 students (99.7% of the total participant) had the internal contamination below the detection limit of the WBC machines. No other radionuclides besides potassium-40 were detected. For students whose measurement results were under the detection limit, assuming a constant daily intake after the nuclear disaster, and from the detection limits of the WBC examination, the maximum annual effective doses from Cs-134 and Cs-137 together were estimated at 0.066, 0.04, and 0.025 mSv/year for children aged from 6 to 7, from 8 to 12, and from 13 to 15 years, respectively. Therefore the calculated additional effective dose from internal radiation exposure ranged from 0.025 to 0.085 mSv/year with the median of 0.04 mSv/year.

### Annual Additional Effective Dose from external radiation exposure

Out of 2,643 measurements, 162 were below the detection limit (i.e. lower than the environmental radiation, which was subtracted from the measured value). The median of the results from the screening programs performed in between 1st June to 31^st^ August 2012, 1^st^ September to 30^th^ November, and 1^st^ December 2012 to 28^th^ February 28^th^ 2013 were 0.17 mSv (range: 0.00–1.48 mSv), 0.17 mSv (range: 0.00–0.99 mSv), and 0.15 mSv (range: 0.00–0.82 mSv), respectively. Therefore the calculated annual additional effective dose from external radiation exposure ranged from 0.00 to 3.45 mSv/year, with the median of 0.66 mSv/year. The levels of external radiation exposure of the three participants whom were detected with internal contamination were 0.92, 1.05, and 1.12 mSv/year, respectively.

### Annual additional effective doses from both internal and external radiation exposure

**Fig 2:**
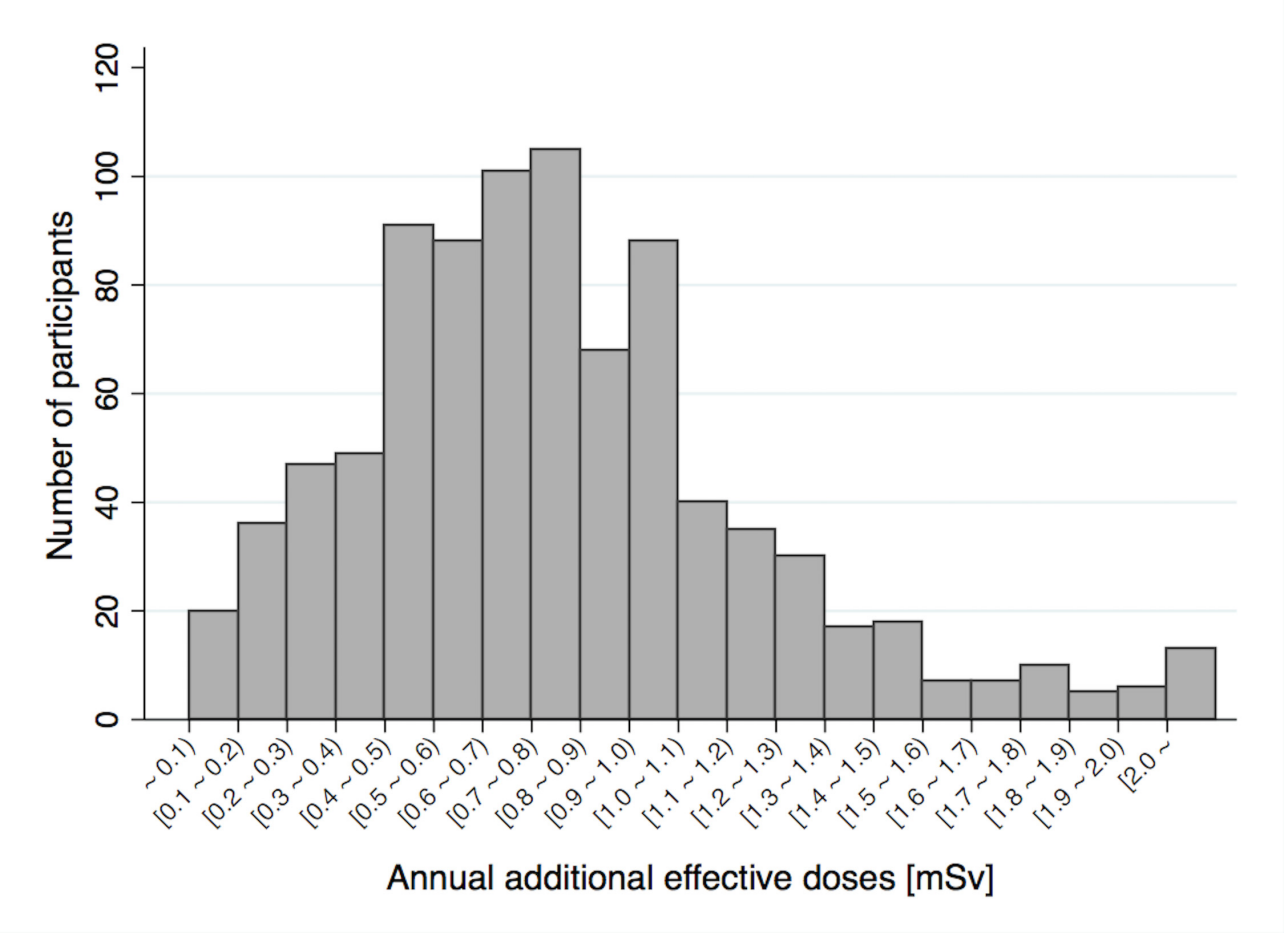
Annual additional effective doses among children in Minamisoma between April 1^st^, 2012 and March 31^st^, 2013.

**Fig 3:**
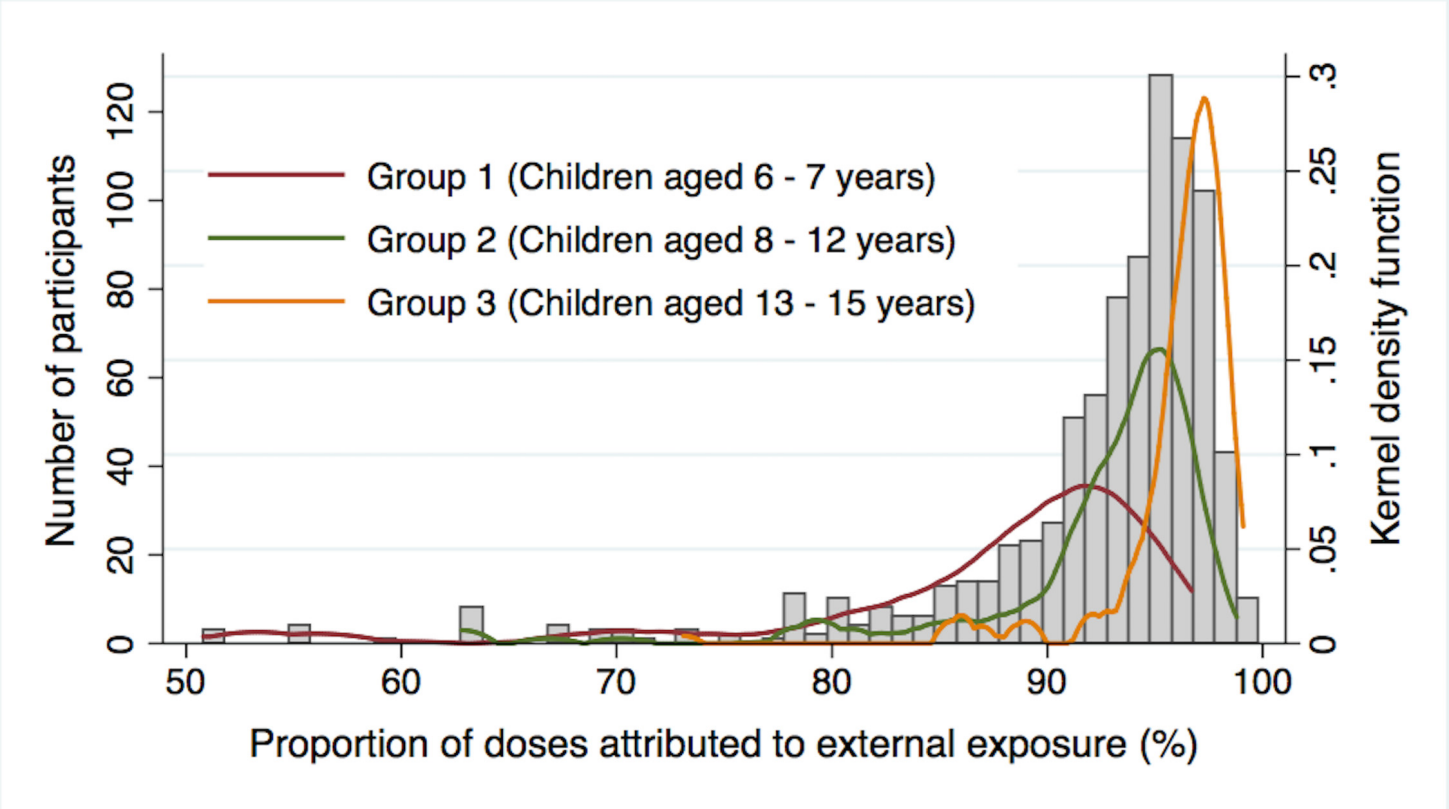
Histogram of the proportion of doses attributed to external exposure with age-group specific kernel density functions. Groups 1, 2, and 3 comprise children aged from 6 to 7 years, from 8 to 12 years, and from 13 to 15 years, respectively. The proportions of doses from external radiation exposure in Group 1, 2, and 3 are 86.3, 89.8, and 94.7%, respectively.

Fig 2 shows the distribution of annual additional effective doses. With the median level of 0.70 mSv/year, the doses ranged from 0.025 to 3.49 mSv/year. Fig 3 is a histogram showing the proportion of doses attributed to external radiation exposure with age-group specific kernel density functions. To enable a clearer visual inspection of the dose distributions, students who had a 'zero' percent contribution of external radiation exposure to the total effective dose were excluded (n = 19). This is because their inclusion could result in an overdispersion on the left-hand side of the distribution. Doses from external radiation exposure accounted for 90.3% of annual additional effective doses.

## Discussion

Previous studies showed that chronic internal contamination was minimal among residents in Minamisoma.[14, 17] However, a little is known about the levels of external radiation exposure. Evaluation of individual external radiation exposure is mandatory for the calculation of doses from chronic radiation exposure and to assess health risks associated with long-term radiation exposure for each resident in the affected areas.

This study showed that the annual additional effective doses 1 to 2 year after the disaster were low enough to minimize health risks associated with radiation exposure among children in Minamisoma. Annual additional effective doses were below 1 mSv/year among 77.9% of children in Minamisoma, which is the accepted standard of radiation exposure in the general public. While the level of soil contamination of Cs-137 ranged from 10k to over 3000k Bq/m^2^ in Minamisoma in November 2011, radiation exposure level among residents in these levels of radiation-contaminated region look controllable. Doses varied among individuals (range: 0.025–3.49 mSv/year) due to lifestyle, area type (urban or rural), and radiation protective factors.[10] It should be noted that the calculated representative dose is not enough to estimate the overall radiation-related health risks among residents. We need to establish a personalized approach so that we can intervene and assist residents with relatively high levels of radiation exposure.

This study also showed that the measured annual effective doses are fairly consistent with the model estimate by UNSCEAR.[9] These authorities estimated that the annual additional effective doses from external and internal radiation exposures would be approximately 0.61 and 0.06 mSv/year, respectively in Minamisoma during this study period, while our results showed that median doses from external and internal radiation exposures were 0.66, and 0.04 mSv/year, respectively. While the Japanese government estimated the additional external effective dose by using the ambient air dose rate, we observed disagreement between the government-led model and the measured additional doses. Doses of measured additional exposure were around three times smaller compared to those derived from government-led modeled additional doses 18 to 30 months after the accident among the study participants in the city of Minamisoma mainly. This was due to the shorter time of outdoor activities and the stronger building shielding effects compared to the model.[24] Estimations used in models tend to be conservative due to the radiation protective aims of the models, the given methodologies, and the limited data sets. However, precise measurements and validations of the estimation methodologies are necessary for evaluating radiation health risks among residents.

This study demonstrated that the annual additional effective doses were predominantly due to doses from external radiation exposure. While annual additional effective doses from internal radiation exposure were calculated below 0.1 mSv/year in all participants, those from external radiation exposure ranged from 0.00 to 3.45 mSv/year. Doses from external radiation exposure accounted for 90.3% of the total dose, even after the calculated maximum doses from internal radiation exposure for those with WBC measurements results of under the detection limits were added. This situation is totally different from the Chernobyl disaster, where doses from internal radiation exposure constituted 40% of total effective doses.[18] This difference might be explained by two reasons. First, countermeasures against food contamination after the Fukushima disaster might have been successful. A criterion set for food inspection was 100 Bq/kg of Cs contaminations, which is relatively strict when compared to those internationally determined criteria. Second, education programs for farmers and local communities intended to prevent internal contamination could have been successful after the Fukushima disaster. While effective doses were low enough for the most of the students in 2012, countermeasures for external radiation exposure including decontamination were useful for lowering the effective doses. However, there is still a chance that the number of individuals with detected internal radiation exposure could rise in the future if local food products are not well regulated as this was the case in Chernobyl. A strict food regulation is therefore one of the important keys in maintaining the low levels of internal exposure.

There are several limitations to this study. First, there is a risk of sampling bias because participation to the screening programs was voluntary. Children tend to be more cautious to prevent radiation exposure, leading to the underestimation of the effective doses. Second, the adherence to study and equipment instructions may differ between individuals in the external screening programs. Third, this study was exclusive to children. The lifestyles of adults differ from those of children, and may thereby result in differences in doses. Fourth, information regarding instruction adherence was not available for the present study, which might lead to potential over- or under-evaluation of the annual effective dose.

## Conclusion

The second year annual additional effective doses among Minamisoma children were low following the Fukushima Daiichi nuclear power plant disaster, even when inter-individual differences were taken into account. Doses from internal radiation exposure were negligible due to the successful control of food contamination. The dose from external radiation exposure accounted for 90.3% of the total effective dose.
